# Evaluation of Neuropathic Pain after Total Knee Arthroplasty: Do Yellow Flags Matter?

**DOI:** 10.3390/jcm12247708

**Published:** 2023-12-15

**Authors:** Danijel Colovic, Alexander Draschl, Patrick Reinbacher, Andrzej Hecker, Gregor Schittek, Stefan Franz Fischerauer, Andreas Leithner, Sebastian Martin Klim, Amir Koutp, Ulrike Wittig, Kevin Brunnader, Andreas Sandner-Kiesling, Patrick Sadoghi

**Affiliations:** 1 Department of Orthopedics and Trauma, Medical University of Graz, Auenbruggerplatz 5, 8036 Graz, Austria; danijel.co.09@gmail.com (D.C.); patrick.sadoghi@medunigraz.at (P.S.); 2 Division of Plastic, Aesthetic and Reconstructive Surgery, Department of Surgery, Medical University of Graz, Auenbruggerplatz 29/4, 8036 Graz, Austria; 3 COREMED—Centre for Regenerative Medicine and Precision Medicine, Joanneum Research Forschungsgesellschaft mbH, Neue Stiftingtalstraße 2, 8010 Graz, Austria; 4 Department of Anesthesiology and Intensive Care Medicine, Medical University of Graz, Auenbruggerplatz 5/5, 8036 Graz, Austria; andreas.sandner@medunigraz.at

**Keywords:** chronic postoperative pain, neuropathic pain, total knee arthroplasty, yellow flags

## Abstract

Up to 20% of total knee arthroplasty (TKA) patients continue to experience chronic postsurgical pain. Various factors have been identified as potential contributors, including so-called “yellow flags”, encompassing symptoms of depression, anxiety, and catastrophizing, which were examined in this study to assess their predictive value concerning functional outcomes after TKA. Methods: Fifty TKA patients were categorized into high-risk and low-risk groups based on clinical assessment, demographic data, medication, and patient-reported outcome measures (DN4, SF-36, WOMAC, NRS, Fibromyalgia Survey Questionnaire, Pain Catastrophizing Scale, and Hospital Anxiety and Depression Scale). Postoperative outcomes within six months after TKA were then compared. Results: Both groups exhibited significant (*p* < 0.001) improvements in all WOMAC and NRS subscales, as well as in the physical function, role physical, pain, and energy/fatigue subdomains of the SF-36 after six months, while the high-risk group showed lower WOMAC scores regarding stiffness (19.0 ± 18.3 vs. 27.2 ± 20.7, *p* < 0.001) and pain (13.5 ± 13.3 vs. 15.1 ± 16.3, *p* = 0.029). The high-risk group showed significantly worse preoperative DN4 scores (1.8 ± 1.3 vs. 3.0 ± 1.1, *p* = 0.002) than the low-risk group, which persisted for one day (2.3 ± 1.2 vs. 3.5 ± 1.5, *p* = 0.005) and six weeks (2.2 ± 1.9 vs. 3.6 ± 2.3, *p* = 0.041) postoperatively. Conclusions: Our results indicate that pre-existing yellow flags contribute to a more challenging early postoperative phase, underscoring the importance of considering individual patient characteristics and psychological factors to optimize TKA outcomes.

## 1. Introduction

As one of the most common debilitating joint illnesses, knee osteoarthritis (OA) poses a significant public health concern, with significant consequences for individuals and health care systems, leading to substantial socioeconomic expenses [1]. Despite the availability of a well-established step-by-step treatment plan comprising physical therapy, medication, and surgical procedures, achieving a long-term solution for OA remains a challenge [2]. The present focus in managing this condition revolves around delaying OA onset primarily through conservative therapy and reserving surgical interventions as a last resort when non-surgical approaches fail to alleviate symptoms [2].

While total knee arthroplasty (TKA) is a well-established and successful treatment option, it is notable that the proportion of dissatisfied TKA patients is significantly higher compared to joint replacement of the hip [3,4]. A particular concern is the occurrence of chronic postsurgical pain (CPSP), which affects approximately 20% of TKA patients, posing a major concern and challenge regarding adequate therapy [5].

The development of CPSP is believed to be primarily influenced by the presence of neuropathic pain, which arises from mechanisms involving both peripheral and central sensitization. These mechanisms affect pain pathways and reduce the efficiency of frequently employed analgesic therapies. In individuals experiencing this neuropathic pain component, the inclusion of co-analgesics in customized treatment plans becomes essential, emphasizing the need for early diagnosis and proper care to improve overall patient outcomes and satisfaction [6]. Several factors, such as demographic characteristics (e.g., female gender and young age), psychological factors (e.g., depression, excessive anxiety, worries, commonly referred to as “yellow flags”), pre-existing chronic pain, and prolonged use of analgesics, are linked to worse treatment outcomes and have been investigated extensively [7,8,9,10,11,12].

Although most results in systematic studies show a correlation at least in some psychological factors like higher depression scores, state of anxiety, and catastrophizing [13,14,15], there remains a lack of sufficient evidence to pinpoint which factors consistently exhibit an association with the development of CPSP [16]. One of the key issues in the topic’s examination is the difficulty in comparison due to the variability of research and assessment methods [13].

By preoperatively identifying patients with an increased risk of developing CPSP, there is an opportunity to implement enhanced postoperative therapeutic measures, including an early multidisciplinary approach [17], encompassing cognitive behavioral therapy [18,19], which can be particularly crucial in cases involving neuropathic pain. The objective of this prospective study was to explore the predictive potential of the mentioned factors for postoperative outcomes following TKA. The findings aim to contribute to the development of a standardized protocol that could be effectively integrated into future clinical practices. The study hypothesis was that the preoperative presence of the aforementioned risk factors would adversely affect the functional outcome, resulting in increased rates of CPSP among patients following TKA.

## 2. Materials and Methods

This prospective study was conducted at a high-volume tertiary hospital, involving a cohort of 50 patients who underwent TKA between March 2021 and July 2021. All procedures were performed by or under direct supervision of one experienced senior surgeon using the ATTUNE™ Knee System by DePuy Synthes. This study was approved by the Institutional Ethical Committee (No. 34-136 ex 21/22) and adhered to the principles as stated in the Declaration of Helsinki.

Before surgery, all patients underwent a comprehensive screening process that included a detailed medical history assessment, clinical examination, and the administration of various patient-reported outcome measures (PROMs) [20,21]. The PROMs used in this study encompassed the Numeric Rating Scale (NRS) [22], the Western Ontario and McMaster Universities Osteoarthritis Index (WOMAC) [23,24], and the Knee Injury and Osteoarthritis Outcome Score (KOOS) [25,26]. Furthermore, an additional five scores were employed to thoroughly capture the biopsychosocial aspects of pain, including the Short Form 36 (SF-36) [27,28,29], the Fibromyalgia Survey Questionnaire (FSQ) [30], the Pain Catastrophizing Scale (PCS) [31,32], the Hospital Anxiety and Depression Scale (HADS) [33], and the Douleur Neuropathique en 4 Questions (DN4) [34,35].

During the initial preoperative examination, demographic data, including date of birth, gender, weight, height, body mass index (BMI), education level (in years), family status, housing situation, occupational status, alcohol consumption, smoking habits, annual gross salary, and date of inpatient admission, were recorded. Injury-specific data encompassed the association of the injury with trauma, duration of illness or onset of pain, affected side, previous attempts at conservative therapy, medication use, type of referral (self-referral, referral from a general practitioner, or referral from a specialist), affected body regions, and waiting time for preoperative anesthetic examination and surgery. Treatment-specific data included surgical details (access, duration, surgeon, and implant) and the type of anesthesia used (general anesthesia or regional anesthesia).

All patients were examined for the presence of allodynia, a characteristic sign of neuropathic pain where the pain is triggered by physiologically non-painful stimulation [36]. Mechanical pressure was applied to the painful area using the blunt and sharp edges of a cotton swab to determine the presence of allodynia. Furthermore, the affected knee’s range of motion (ROM) was measured using a goniometer in both active and passive movements.

A council of two senior anesthesiologists and two orthopedic surgeons subsequently reviewed the obtained data to categorize the patients into a high-risk (n = 20) and low-risk group (n = 30). A standardized pain protocol was used in both groups: From the day of surgery until the second postoperative day, all patients received 250 mL neodolpasse (75 mg diclofenac/30 mg orphenadrine) intravenously (i.v.) every 12 h, 1 g metamizole i.v. every 6 h, and 7.5 mg piritramide i.v. every 12 h as basic medication. From the third postoperative day, medication was administered orally and switched to 50 mg diclofenac every 8 h, 500 mg metamizole every 6 h, and 2 mg slow-release hydromorphone every 12 h (for 3 days). For severe pain, 7.5 mg piritramide i.v. could also be administered up to 4 times a day until the second postoperative day, respectively, 1.3 mg hydromorphone per os (p.o.) as well as 500 mg paracetamol p.o. up to 4 times a day after the third postoperative day as an on-demand medication. In case of contraindication/intolerance to diclofenac or metamizole, 1 g paracetamol i.v. was prescribed every 8 h until the second postoperative day, respectively, 500 mg every 6 h from the third postoperative day. Further home medication was adjusted depending on the individual pain situation, whereby a symptom-oriented reduction in pain medication was possible from the third postoperative day.

Follow-up examinations were conducted on the second postoperative day, the day of discharge, as well as six weeks, three months, and six months after TKA.

### Statistical Analysis

All statistical analyses were performed using IBM^®^ SPSS^®^ (Statistics 27, Armonk, North Castle, NY, USA). Determined factors are presented as absolute and relative frequencies and means and standard deviations (SD). Partial eta-squared (η^2^) was used for estimation of effect size (η^2^ = 0.01 indicates a small effect, η^2^ = 0.06 indicates a medium effect, η^2^ = 0.14 indicates a large effect). The Kolmogorov–Smirnov test was used to analyze data with a parametric distribution, while continuous data were analyzed using two-tailed *t*-tests or non-parametric Mann–Whitney U tests. A repeated measures analysis of variance (ANOVA) was used to assess within-subject effects. Further analyses included logistic regression on neuropathic pain. The logistic regression model fitted with the covariates of age, gender, and BMI. Tendency above 1 in odds ratio (OR) stands for higher risk of neuropathic pain.

The results were only considered if sphericity was determined by a non-significant Mauchly-W test (*p* > 0.05). However, if the Mauchly-W test was significant (*p* < 0.05), correction factor epsilon (ε) was used to adjust the degrees of freedom. The Greenhouse–Geisser correction was employed if its epsilon was less than 0.75, and the Huynh–Feldt correction if it was greater than 0.75. Bonferroni-corrected pairwise comparisons were utilized to further discriminate when significant differences between within-subject variables occurred. According to a sample size calculation, a sample of at least 50 patients would be required [37]. A two-tailed *p*-value of ≤0.05 was considered significant.

## 3. Results

This study involved a total of 50 patients, with 31 females (62%) and 19 males (38%) included in the analysis. Among the participants, 20 patients (40%) were classified as high risk (Group I), with an imbalanced gender distribution of 4 males (20%) and 16 females (80%). The low-risk group (Group II) consisted of 30 patients (60%), with an equal gender distribution of 15 females (50%) and 15 males (50%). The difference in gender distribution was found to be statistically significant (*p* = 0.032), whereby significantly more women were found in the high-risk group (Table 1). All the other demographic features were comparable in both groups, as outlined in Table 1.

### 3.1. Preoperative Outcomes

The preoperative evaluation revealed that the high-risk group had worse scores than the low-risk group regarding the HADS total (*p* = 0.012), HADS anxiety (*p* = 0.038), and HADS depression (*p* = 0.008). The high-risk group showed significantly worse preoperative scores in FSQ (U = 177.5, Z = −2.453, *p* = 0.014) and DN4 (U = 151, Z = −3.026, *p* = 0.002) (Table 1). Significant interaction effects were found between the low-risk and high-risk groups in the WOMAC subscales of pain (*p* = 0.029) and stiffness (*p* < 0.001), with significantly higher scores observed in the high-risk group preoperatively (Table 2). Moreover, no further significant differences were observed in the PCS, KOOS, in other subscales of the WOMAC, in NRS (maximum, rest and activity), and SF-36 scores (Table 1 and Table 2).

### 3.2. Neuropathic Pain

The high-risk group showed significantly worse DN4 scores (low-risk group: 2.3 ± 1.2 vs. high-risk group: 3.5 ± 1.5, U = 162, Z = −2.789, *p* = 0.005) and FSQ scores (low-risk group: 2.1 ± 2.3 vs. high-risk group: 2.9 ± 1.6, U = 195.5, Z = −2.1, *p* = 0.036) after one day postoperatively. Six weeks postoperatively, the high-risk group presented significantly worse DN4 outcomes (low-risk group: 2.2 ± 1.9 vs. high-risk group: 3.6 ± 2.3, U = 198, Z = −2.044, *p* = 0.041), whereas no significant difference could be found in the outcomes of FSQ (low-risk group: 2.1 ± 1.6 vs. high-risk group: 2.9 ± 2.3, U = 238, Z = −1.246, *p* = 0.213). The logistic regressions adjusted for age (OR = 0.960, *p* = 0.271), gender (OR = 0.232, *p* = 0.08), and BMI (OR = 0.854, *p* = 0.067) were all non-significant.

### 3.3. Postoperative Outcomes after 6 Months

Our findings demonstrated significant improvements in the SF-36 subscales of physical function, role physical, energy/fatigue, and pain, as well as in all WOMAC and NRS subscales in both groups six months postoperatively (Table 2). However, no further significant differences were observed in other subscales of the SF-36 (role emotional, emotional well-being, social functioning, general health), as well as in range of motion (ROM). In the SF-36 subscale of physical function, role physical, and pain, significantly higher scores were achieved after 3 (*p* < 0.001, *p* = 0.031, *p* < 0.001, respectively) and 6 months (*p* < 0.001, respectively) postoperatively. Whereas in the subscale energy/fatigue, significantly higher scores could be found after 3 months (*p* = 0.031), but not after 6 months (*p* = 0.052) after TKA. A pairwise comparison revealed significant improvements in all NRS subscales after 3 (maximum: *p* < 0.001, rest: *p* = 0.032, activity: *p* < 0.001) and 6 months (maximum: *p* < 0.001, rest: *p* = 0.002, activity: *p* < 0.001) (Table 2).

### 3.4. Postoperative Outcomes between Low-Risk Group and High-Risk Group

After 3 months and 6 months, the high-risk group presented significantly lower pain and stiffness scores (*p* < 0.001, respectively), but there was no significant difference between 3 months and 6 months (pain: *p* = 0.896, stiffness: *p* = 1.000). Moreover, no further significant differences were observed in other subscales of the WOMAC, as well as in the subscales of the NRS and SF-36 scores (Table 2).

## 4. Discussion

In this prospective study, we aimed to comprehensively analyze the impact of known risk factors on patient outcomes following TKA. Overall, both the high-risk and low-risk groups showed satisfactory results with low complication rates. However, it is important to note that patients in the high-risk group initially presented with worse outcomes in pain, functional ability, health-related quality of life, and a stronger neuropathic pain component (Table 1). Interestingly, these differences diminished over the course of the study, and in certain parameters, such as the pain and stiffness subscales of the WOMAC, the high-risk group even demonstrated significantly better values in comparison to the low-risk group at the final 6-month follow-up (Table 2). Our findings indicate that preoperative yellow flags may contribute to a more challenging early postoperative phase after TKA, as indicated in a previous study [38].

To see if our grouping would reveal significant differences that could potentially limit further data analysis, we compared the preoperative values of various variables between the groups, listed in Table 1. We found significant gender differences, with a higher proportion of women in the high-risk group. This disparity can be partly explained by the factors used for classification. Despite controversy regarding the gender distribution of neuropathic pain, the literature suggests that women tend to be more susceptible to pain and depression, which are symptoms assessed in the PROMs used for classification [39,40,41]. According to a meta-analysis by Abate et al., male patients are 63% less likely to experience depression than female patients [42]. Additionally, women tend to have a higher possibility of developing knee osteoarthritis in general, which may have contributed to these findings [2].

Regarding the hypothesized risk factors for worse outcomes, we found significant disparities between the high-risk and low-risk groups in various PROMs. Particularly, there were significant differences between the groups regarding the HADS total, HADS subscales for depression and anxiety, as well as the FSQ and DN4 questionnaires. In these PROMs, the high-risk group consistently showed significantly worse values compared with the low-risk group. The PCS showed marginally higher values in the high-risk group, but this difference did not reach statistical significance (Table 1). As the grouping was based in part on these PROMs, these results were to be expected and did not restrict further data analysis.

Ali et al. accompanied 186 patients over 4 years and concluded that patients with preoperative anxiety or depression were six times more likely to be unsatisfied with their outcome [8]. Conversely, Ortiz et al. reported contrasting findings, suggesting that preoperative anxiety and depression assessed using the HADS had no impact on the functional outcomes of TKA. Interestingly, patients with higher levels of anxiety experienced greater pain relief than those with higher levels of depression [43]. However, the relationship between knee pain, anxiety, and depression is complex, and TKA can be a beneficial intervention for improving both conditions, as highlighted by Blackburn et al. [44].

The role of pain catastrophizing as a predictor for postoperative pain following TKA has been examined in previous studies. On the one hand, Birch et al. found that patients with high PCS scores prior to surgery experienced more pain, worse overall health, and lower physical function one year postoperatively [12]. On the other hand, Hovik et al. observed no association between preoperative pain catastrophizing and postoperative pain during the same time period [45].

The literature presents conflicting evidence concerning the role of neuropathic pain in the development of CPSP and its impact on outcomes following TKA. Beloeil et al. [9] reported that severe neuropathic pain in the initial postoperative days significantly increases the likelihood of CPSP. Similarly, Hasegawa et al. [46], assessing neuropathic pain with the DETECT questionnaire, found a potential association between CPSP development and the presence of pre-existing neuropathic pain. However, Fitzsimmons et al. [47], using a methodology comparable to our approach, concluded that while suspected neuropathic pain may increase pain levels, catastrophizing, and depressive symptoms, it may not be a significant predictor of TKA outcomes at the 6-month follow-up. These conflicting findings highlight the need for further research to better understand the complex interplay between neuropathic pain, CPSP, and TKA outcomes.

In our study, patients in the high-risk group presented higher DN4 scores at all measurement points, while the significance slightly decreased over time (preoperative: 1.8 ± 1.3 vs 3.0 ± 1.1, *p* = 0.002, first day postoperative: 1.8 ± 1.3 vs. 3.0 ± 1.1, *p* = 0.005, six weeks postoperative: 2.2 ± 1.9 vs. 3.6 ± 2.3, *p* = 0.041). Our findings align with a study that grouped TKA patients into two groups according to their PCS (physical component score of the SF-36 survey), revealing that a higher proportion of individuals with PCS ≥ 50 exhibited higher DN4 scores one year after TKA [48].

We used SF-36, WOMAC, and NRS to quantify our claims about clinical outcomes and quality of life. In both groups, we found a significant improvement in the results of several SF-36 subscales, including physical function, role physical, energy/fatigue, and pain, all NRS subscales (maximum, rest, activity), as well as significant improvements in the WOMAC index over a time course of 6 months after TKA (Table 2). These results support previous findings by indicating that TKA has a strong favorable impact on life quality, functional ability, and pain across both groups of our population [49,50].

Throughout the trial, there was a significant interaction effect between the pain and stiffness subscales of the WOMAC, with lower values in the high-risk group after 3 and 6 months postoperatively (Table 2). These findings contradict the prevailing beliefs in the literature, which typically suggest that the high-risk group would experience worse outcomes [7,10,11,50]. The lower scores in the high-risk group indicate that other factors might be influencing the outcomes in this specific subpopulation, again highlighting the complexity of the relationship between the assessed risk factors and TKA outcomes. We interpret this against the backdrop of a divergence between the WOMAC pain and stiffness scores, tailored to joint-specific aspects, and NRS and SF-36 scores, encompassing a broader pain spectrum, while acknowledging the subjective nature of pain [51]. This implies a nuanced shift in factors influencing pain perception in high-risk patients, suggesting that their pain experience may extend beyond knee-specific issues to involve other body regions.

Furthermore, Lee et al. investigated 142 patients over the timeframe of one year, to measure the effects of nocturnal pain (72% of patients), neuropathic pain (15% of patients), and depressive disorders (13% of patients) on the outcome of TKA. They concluded that patients with night pain had significantly worse functional and quality of life scores preoperatively, but that these negative effects disappeared after one year and had no influence on the outcome [52]. Their results indicate that the influence of preoperative neuropathic pain, pain at night, and depressive disorder on TKA outcomes may vary over time and require long-term assessment.

## 5. Limitations

This study had several limitations that should be considered. Firstly, the sample size was rather small, potentially influencing the statistical power and limiting the generalizability of the findings to larger populations. Secondly, the relatively short follow-up period of 6 months, or only 6 weeks in the case of the DN4 assessments, might not adequately reflect long-term outcomes and the potential influence of the investigated parameters on chronic postsurgical pain beyond this timeframe. Moreover, the subjective classification of patients into high-risk and low-risk groups as well as the subjective perception of pain itself introduces further potential for bias and subjectivity, making comparisons with previous studies challenging. Furthermore, the lack of a control group limits the direct comparison of outcomes between the high-risk and low-risk groups with patients who did not have TKA. A control group would have been a useful reference point for assessing the impact of yellow flags on TKA outcomes.

We ensure transparency and provide important context for the interpretation of this study’s results by acknowledging these limitations. These limitations also highlight areas for future research, such as larger sample size studies, more objective classification methods, appropriate control groups, and extending the follow-up period to assess the long-term impact of yellow flags on TKA outcomes.

## 6. Conclusions

Predicting the outcome of patients undergoing TKA based on preoperative factors is a complex task. We found that the presence of yellow flags (e.g., symptoms of depression, anxiety, and catastrophizing) and a neuropathic pain component contributed to a more difficult early postoperative phase, with worse functional outcomes, higher pain levels, and lower health-related quality of life. These differences diminished over the course of this study, allowing patients in the high-risk group to derive the greatest joint-specific outcome improvement from this intervention. These findings emphasize the importance of considering individual patient characteristics and psychological factors in optimizing the outcomes of TKA. Further research exploring predictive factors, including yellow flags, and refining patient selection criteria is required to enhance personalized care and maximize the benefits of TKA for all patients.

## Figures and Tables

**Table 1:** Differences in patient data and preoperative PROMs between the high-risk group (Group I) and low-risk group (Group II).

		Group I (High Risk)	Group II (Low Risk)	*p*-Value
		n = 20	n = 30	
Age (mean ± SD)		67.8 ± 10.5	66.8 ± 9.5	0.719 ^a^
Gender (%)	Female	16 (80)	15 (50)	**0.032** ^b^
	Male	4 (20)	15 (50)	
BMI (mean ± SD)		29.7 ± 4.3	33.2 ± 6.1	0.056 ^a^
ASA (%)	1	1 (5)	2 (6.7)	0.978 ^b^
	2	8 (40)	13 (43.3)	
	3	10 (50)	14 (46.7)	
	4	1 (5)	1 (3.3)	
HADS (mean ± SD)	Total	13.2 ± 7.2	8.1 ± 6.3	**0.012** ^a^
	Anxiety	8.2 ± 4.4	5.7 ± 4.0	**0.038** ^a^
	Depression	5.0 ± 3.5	2.4 ± 2.9	**0.008** ^a^
PCS (mean ± SD)		16.9 ± 10.5	11.1 ± 10.4	0.061 ^a^
NRS (mean ± SD)	Maximum	6.9 ± 2.0	6.0 ± 2.2	0.188 ^a^
	Rest	2.5 ± 2.5	2.1 ± 2.0	0.625 ^a^
	Activity	4.9 ± 2.3	4.7 ± 2.4	0.867 ^a^
KOOS (mean ± SD)	Total	97.0 ± 27.2	83.0 ± 26.1	0.075 ^a^
	Pain	19.8 ± 6.1	16.4 ± 6.6	0.073 ^a^
	Symptoms	13.7 ± 5.7	11.4 ± 4.4	0.127 ^a^
	Activity	35.6 ± 12.2	29.7 ± 11.9	0.097 ^a^
	Functionality	17.1 ± 3.1	15.6 ± 4.5	0.198 ^a^
	Quality of life	10.9 ± 2.3	9.9 ± 3.2	0.232 ^a^
FSQ (mean ± SD)		4.1 ± 2.2	2.4 ± 2.0	**0.014** ^c^
DN4 (mean ± SD)		3.0 ± 1.1	1.8 ± 1.3	**0.002** ^c^

^a^ Unpaired *t*-test, ^b^ Pearson’s chi-squared-test, ^c^ Mann–Whitney U test. SD: standard deviation; PROM: patient-reported outcome measurements; BMI: body mass index; ASA: American Society of Anesthesiologists; HADS: Hospital Anxiety and Depression Scale; PCS: Pain Catastrophizing Scale; NRS: Numeric Rating Scale; KOOS: Knee Injury and Osteoarthritis Outcome Score; FSQ: Fibromyalgia Survey Questionnaire; DN4: Douleur Neuropathique en 4. Bold values indicate significant *p*-values.

**Table 2:** Results of repeated measures analysis of variance (ANOVA) of the SF-36, WOMAC, NRS, and ROM questionnaires for high-risk group (Group I) and low-risk group (Group II).

		Group I (High Risk) n = 20			Group II (Low Risk) n = 30			*p*-Value (Within Subject)	Effect Size (η^2^)	*p*-Value (Interaction Effect)	Effect Size (η^2^)
		Preoperative	3 Months	6 Months	Preoperative	3 Months	6 Months				
SF-36 (mean + SD)	Physical function	32.1 ± 17.7	60.5 ± 21.7	67.0 ± 19.5	46.5 ± 28.3	56.1 ± 21.5	66.1 ± 18.0	**<0.001**	0.373	0.076	0.085
	Role physical	15.3 ± 27.0	32.9 ± 31.6	52.9 ± 37.4	29.2 ± 32.8	47.5 ± 32.7	59.4 ± 37.5	**<0.001**	0.249	0.796	0.006
	Role emotional	50 ± 45.5	60.4 ± 42.5	64.6 ± 46.3	72.2 ± 38.9	81.9 ± 32.6	77.8 ± 30.6	0.294	0.032	0.793	0.006
	Energy/fatigue	42.1 ± 19.5	52.6 ± 16.7	59.4 ± 20.7	58.8 ± 22.6	66.5 ± 15.3	61.9 ± 19.0	**0.011**	0.119	0.108	0.057
	Emotional well-being	62.4 ± 21.4	70.6 ± 19.7	70.8 ± 19.6	79.2 ± 17.1	79.8 ± 13.1	76.7 ± 18.8	0.24	0.07	0.115	0.054
	Social functioning	69.1 ± 31.3	86.8 ± 15.0	91.2 ± 15.8	90.1 ± 70.7	89.1 ± 13.9	84.4 ± 19.9	0.414	0.019	0.208	0.041
	Pain	34.7 ± 22.3	59.0 ± 15.9	61.0 ± 18.9	36.1 ± 20.1	67.3 ± 17.8	69.5 ± 20.3	**<0.001**	0.526	0.62	0.011
	General health	57.0 ± 18.5	62.9 ± 19.0	62.1 ± 19.9	66.3 ± 17.6	71.8 ± 16.0	67.3 ± 19.2	0.102	0.057	0.707	0.009
WOMAC (mean + SD)	Total	41.9 ± 17.0	12.9 ± 10.4	14.8 ± 11.5	40.5 ± 20.3	17.7 ± 14.6	18.2 ± 14.6	**<0.001**	0.508	0.522	0.06
	Pain	48.4 ± 18.5	11.9 ± 10.6	13.5 ± 13.3	35.0 ± 18.3	12.5 ± 11.0	15.1 ± 16.3	**<0.001**	0.588	**0.029**	0.061
	Stiffness	58.3 ± 25.8	19.0 ± 17.1	19.0 ± 18.3	34.3 ± 23.4	23.5 ± 21.0	27.2 ± 20.7	**<0.001**	0.302	**0.002**	0.191
	Activity	41.2 ± 16.8	12.5 ± 10.6	14.8 ± 12.4	40.7 ± 22.4	18.4 ± 17.5	18.0 ± 15.0	**<0.001**	0.46	0.587	0.55
NRS (mean + SD)	Maximum	6.9 ± 2.1	3.5 ± 1.5	3.7 ± 2.5	6.2 ± 2.0	2.6 ± 2.4	2.2 ± 2.4	**<0.001**	0.53	0.592	0.013
	Rest	2.5 ± 2.6	1.5 ± 1.1	0.7 ± 1.1	2.0 ± 2.0	0.8 ± 1.3	0.9 ± 1.8	**0.001**	0.184	0.451	0.264
	Activity	4.7 ± 2.4	2.3 ± 1.4	2.6 ± 1.7	4.6 ± 2.4	2.5 ± 2.4	2.3 ± 2.1	**<0.001**	0.308	0.744	0.001
ROM (mean + SD)		111.8 ± 18.8	108.0 ± 11.5	110.6 ± 14.3	103.8 ± 16.6	96.7 ± 24.6	105.0 ± 19.4	0.183	0.043	0.691	0.021

SD: standard deviation; SF-36: Short Form 36; WOMAC: Western Ontario and McMaster Universities Osteoarthritis Index; NRS: Numeric Rating Scale; ROM: range of motion. Bold values indicate significant *p*-values.

## Data Availability

Detailed data supporting the results are available from the authors.
